# Progress in Ophthalmology

**Published:** 1903-03-28

**Authors:** 


### Progress in Ophthalmology.

**Mercury in Perforating Wounds of the Globe.**—Dr. Berry,[1] in the *Edinburgh Medical Society's Journal*, draws attention to the practice of Dr. Schirmer of Griefswald, in the treatment of perforating wounds of the globe. Dr. Schirmer having made up his mind that "mercury is the sheet anchor of the surgeon in sympathetic ophthalmia" following injuries of the eyeball says, why not administer the mercury at once, and not wait for the signs and symptoms of sympathetic mischief to manifest themselves? He has therefore for some time past tried this treatment, and administered mercury in every case of infected perforating wounds of the eye, and says, after acting in this way, he began to notice that he delayed the excision of the eyes, longer and longer, and under patient and long continued treatment, the eyes have regained or retained useful vision in many cases. Also that in six years he has not had one case of sympathetic mischief. His watchwords of procedure with regard to mercury are quoted as being "Early, Plenty, and Long." As regards the first point—all the cases which did not come to him till four days after the injury were enucleated, but of those which came before the second day, fully two-thirds recovered. Therefore the treatment should be begun immediately. When a case comes for treatment Schirmer asks himself not how severe the case is, or how much mercury will it need to cure it, but how much mercury will the patient bear? In many of the cases, to start the results of the mercurial treatment quickly, intramuscular injections of biniodide of mercury were given, but the principal method was by inunction with unguentum hydrargyri. Of this ointment he uses for a man from 8 to 9 grammes daily; for a woman 6 to 8 grammes, and for a child 1 to 3 grammes, according to the age of the patient. At the same time, for the first day or two, the patients had subconjunctival injections of corrosive sublimate, and this was soon changed for salt solution, which, although not so efficacious, was better tolerated. The patient was kept in bed and well fed, and great care was taken to keep the mouth in good order. The mercury was with these precautions very well borne. As to the duration of treatment. A few times it happened that after the inunction had been stopped, in the belief that the symptoms had finally ceased, they reappeared, and were much more difficult to deal with than they were previously. His results are summarised thus:—Enucleation was performed in 31 per cent., 21 per cent. recovered with less vision than 1-10, while 48 per cent. recovered with useful vision.

**Ichthyol in Trachoma.**—Concerning trachoma and its treatment, Berry[2] very pertinently remarks: When a large number of different modes of treatment are suggested for any disease, the probabilities are either that it is a very intractable malady, or that it has a strong disposition towards spontaneous recovery. In the first of these directions trachoma is no exception to this general rule, for it resists more or less successfully any and every method of attack. Its course, however, can be modified, and the condition of the patient much improved, by judicious treatment. Eberson pointed out the essentials of treatment of trachoma to be an application that will constrict the dilated vessels, remove the infiltration and thickening of the conjunctiva, and alleviate the various subjective symptoms, particularly the pain, lacrimation, and photophobia. Ichthyolate of ammonia in his hands did all this in a 50 per cent. solution, and did it better than anything he had previously employed. Darier used it undiluted, but this does not seem a wise plan. Denti thought the acute forms were more influenced than the chronic, and its action was especially valuable in cases showing superficial ulceration with pannus. The first effect of application in 50 per cent. strength painted on the everted lids and washed off with distilled water was a slight burning sensation which quickly passes off, and is succeeded by relief from photophobia blepharospasm and pain, and this relief is not merely transitory. The vessels of the pannus shrivel up under its use, and the corneal opacity clears. It is considered to be chiefly in virtue of its deoxidising effect upon tissues that its antiseptic properties are due; it cuts off the supply of nutriment for the micro-organisms rather than directly kills them. Bioletti warmly recommends its employment.

[1] Edin. Med. Jour., June 1902. [2] Ibid.

(*To be continued.*)

